# Estimating a population cumulative incidence under calendar time trends

**DOI:** 10.1186/s12874-016-0280-6

**Published:** 2017-01-11

**Authors:** Stefan N. Hansen, Morten Overgaard, Per K. Andersen, Erik T. Parner

**Affiliations:** 1 Section for Biostatistics, Aarhus University, Bartholins Allé 2, Aarhus C, DK-8000 Denmark; 2 Section of Biostatistics, University of Copenhagen, Øster Farimagsgade 5, Copenhagen K, DK-1014 Denmark

**Keywords:** Cumulative incidence, Time to event, Dependent censoring, Stratification, Time trends

## Abstract

**Background:**

The risk of a disease or psychiatric disorder is frequently measured by the age-specific cumulative incidence. Cumulative incidence estimates are often derived in cohort studies with individuals recruited over calendar time and with the end of follow-up governed by a specific date. It is common practice to apply the Kaplan–Meier or Aalen–Johansen estimator to the total sample and report either the estimated cumulative incidence curve or just a single point on the curve as a description of the disease risk.

**Methods:**

We argue that, whenever the disease or disorder of interest is influenced by calendar time trends, the total sample Kaplan–Meier and Aalen–Johansen estimators do not provide useful estimates of the general risk in the target population. We present some alternatives to this type of analysis.

**Results:**

We show how a proportional hazards model may be used to extrapolate disease risk estimates if proportionality is a reasonable assumption. If not reasonable, we instead advocate that a more useful description of the disease risk lies in the age-specific cumulative incidence curves across strata given by time of entry or perhaps just the end of follow-up estimates across all strata. Finally, we argue that a weighted average of these end of follow-up estimates may be a useful summary measure of the disease risk within the study period.

**Conclusions:**

Time trends in a disease risk will render total sample estimators less useful in observational studies with staggered entry and administrative censoring. An analysis based on proportional hazards or a stratified analysis may be better alternatives.

**Electronic supplementary material:**

The online version of this article (doi:10.1186/s12874-016-0280-6) contains supplementary material, which is available to authorized users.

## Background

The general risk of a disease or psychiatric disorder is commonly estimated in epidemiological cohort studies using various measures of frequency. Among the most common measures are the age-specific cumulative incidence and the prevalence at a given point in time. The age-specific cumulative incidence is the probability of contracting the disease before a given age and is often used to describe changes in disease risks over time and to compare disease risks across geographical regions. As an example, studies from around the world have altogether demonstrated a substantial rise in the risk of autism over time [10]. Ultimately, disease risk estimates are used in health care decision-making and in the allocation of research funding.

Cumulative incidence estimates are often derived in cohort studies in which individuals are included during a pre-specified period and subsequently monitored for the disease until an administrative end of follow-up date. This type of design with staggered entry and administrative censoring is very common within health sciences. Two classic examples are clinical trials with patients enrolled over time and retrospective follow-up studies with data from national health registries. In this type of study we will distinguish between two types of censoring. The first being administrative censoring (or end-of-study censoring) which happens when an individual is followed until the last day of the study period without getting the disease. The second type is loss-to-follow-up censoring which is when an individual is lost to follow-up before the administrative end of follow-up date, e.g., due to emigration or unwillingness to further participate in the study.

Due to differences in the length of follow-up, time-to-event methods are called for in the statistical analysis. Methods for analyzing right-censored data have been available for decades, for example, the age-specific cumulative incidence may be unbiasedly estimated (at least approximately) by one minus the Kaplan–Meier estimator [13] assuming no competing risks or by the Aalen–Johansen estimator [1, 3] if competing risks are present. Both estimators do however require that censoring is independent [3]. An independent censoring mechanism is thus a key assumption in time-to-event analysis as it allows results obtained from a censored sample to be generalized to the target population without censoring. Intuitively, it may be thought of as the requirement that subjects who remain at risk are representative for the sample without censoring with respect to their disease experience at any given time. In a general time-to-event setting it is however not possible, based on the observed data, to determine whether a censoring mechanism is independent or not [21, 22].

Instead of reporting the whole Kaplan–Meier-curve (or Aalen–Johansen-curve in the competing risks scenario), one often settles for the cumulative incidence at just one or a few ages. This might be the cumulative incidence estimate at the largest observed age corresponding to the rightmost point of the Kaplan–Meier curve. This we will refer to as the end of follow-up cumulative incidence estimate. It might also be the cumulative incidence at 100 years which may be interpreted as the probability of contracting the disease within a lifetime and is thus often referred to as the lifetime risk. Examples of this practice are many and not confined to a specific research field [7–9, 15–17, 19, 20].

We will say that calendar time trends are present in a disease risk if the risk depends on the time of entry into the study. Calendar time trends are for instance seen in many psychiatric disorders [5] since diagnostics within psychiatry have changed considerably over time. In this paper, we show that in a cohort study with staggered entry and administrative censoring, the hypothesis of independent censoring of the administrative part of the censoring mechanism is equivalent to the hypothesis of no calendar time trends in the disease risk. The hypothesis of no calendar time trends may easily be assessed in a stratified analysis with strata given by time of entry into the study.

We argue that, under calendar time trends, the total sample Kaplan–Meier and Aalen–Johansen estimators will not describe the general risk of the target population in any useful way. Even though an analyst may be aware of calendar time trends, he or she may think that these total sample estimators will estimate the mean age-specific cumulative incidence or the mean age-specific cumulative incidence within the study period. Using different examples, we will show that this is not the case. In a simulated example, we show that if calendar time trends are given by a proportional hazards model across strata, then total sample estimators will not estimate the mean cumulative incidence across strata. Instead we show how the proportionality of hazards may be used to extrapolate the cumulative incidence within each stratum and subsequently combined into an estimate of the mean cumulative incidence. When this proportionality assumption is unreasonable, we instead advocate for presenting the results from a stratified analysis as the main finding. In another example focusing on psychiatric disorders in Denmark, we show that total sample estimators do not estimate the mean cumulative incidence within the study period either. We argue instead that a weighted average of the end of follow-up estimates from a stratified analysis may be a useful summary measure of the disease risk within the study period and may therefore be used in cross-country comparisons of the disease risk.

## Methods

We will primarily be interested in estimating an event risk in a scenario without delayed entry and with no or limited competing risks. This will particularly be the case in studies where individuals enter at birth and where we consider the risk of a disease or psychiatric disorder at a relatively young age so that death may be disregarded as a competing event. For this reason, our main focus will be on the Kaplan–Meier estimator but our recommendations are equally relevant to the more general setting with delayed entry and competing risks. We will however briefly introduce the Aalen–Johansen estimator which should be used when competing risks cannot be disregarded.

### The independent censoring assumption

Let *T* be a random variable measuring the time from entry at the date *E* to the event of interest. Suppose individuals are under observation from entry until a fixed administrative censoring date *D*. Let *C*_1_ denote the censoring time corresponding to the administrative censoring, i.e., *C*_1_=*D*−*E*, and let *C*_2_ denote the loss-to-follow-up censoring time that may occur prior to the administrative censoring. Both censoring mechanisms may prevent us from observing *T*, that is, we observe $\tilde {T}=T\wedge C$ and *Δ*=**1**_*T*≤*C*_ with *C*=*C*_1_∧*C*_2_.

Independent censoring is known to hold if *T* is stochastically independent of *C* [3] but the assumption is generally not testable based on the observed data [21, 22]. However, for a study with staggered entry and administrative end of follow-up, independence of the administrative part of the censoring is equivalent to the absence of calendar time trends which is in fact testable. To see this, note that, since *D* is deterministic, independence between *T* and *C*_1_=*D*−*E* is the same as independence between *T* and *E* meaning no calendar time trends in the event risk. The assumption of no calendar time trends in the event risk can easily be assessed in a stratified analysis by groups of *E* and we let *B* denote that group variable. Although it is not possible to determine if the loss-to-follow-up censoring *C*_2_ is independent of *T* based on the observed data, we do however note that in many practical examples, the administrative censoring will make up the majority of all censorings meaning that independence between *T* and *C* will in large be justified if we have independence between just *T* and *C*_1_.

In a later example, we will consider the sample consisting of all individuals born in the period 1980–2007 in Denmark with the event of interest being the diagnosis of some psychiatric disorder and with an administrative end of follow-up on December 31, 2013. In this example, *E* is the date of birth, *D* is December 31, 2013, and hence *C*_1_ is the age of the individual at December 31, 2013. The variable *C*_2_ will in this example be the age at emigration to another country and *C* the minimum of the two censoring ages. Censoring due to emigration will in our example only make up about 4% of all censoring meaning that independent censoring is roughly a matter of the administrative censoring *C*_1_ being independent of *T*. When we later return to this example, *B* will denote 1-year birth groups and we will assess calendar time trends by comparing estimated age-specific cumulative incidence curves across the 28 strata given by *B*.

In the following we consider a scenario where *T* is *not* independent of *C* but in which it is possible to divide the study sample into, say, *k* strata given by the random variable *B* such that censoring is (approximately) independent within each stratum. This is exactly the case in a study with staggered entry, administrative censoring and an independent loss-to-follow-up censoring if strata is given by time of entry. Let *n*_*i*_ denote the number of individuals and *t*_*i*_ the time at end of follow-up in the *i*th stratum, respectively, so that *π*_*i*_=*P*(*B*=*i*) is estimated by *n*_*i*_/*n* with $n=\sum _{i} n_{i}$ being the number of individuals in the total sample.

### The at-risk set in a Lexis diagram

**Fig. 1:**
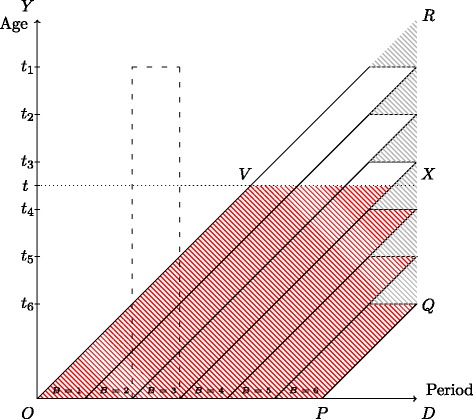
Lexis diagram showing individuals entering the study in the period between *O* and *P* with end of follow-up given by *D*. The individuals are grouped into six strata (*B*) by their time of entry with *t*_1_,…,*t*_6_ denoting the common end of follow-up age for each stratum. The *grey*, hatched area denotes the data that we need to drop to get rid of administrative censoring within each stratum. The resulting data available before age *t* is the *red*, hatched area

The at-risk set in a study with staggered entry and administrative censoring is nicely displayed in a Lexis diagram. Suppose individuals are recruited into the study at birth during the period determined by the two dates *O* and *P* and subsequently followed until the end of follow-up date *D*. Let *B* be the variable that divides this recruitment period into, say, *k*=6 strata of equal length. This setup is illustrated in the Lexis diagram below (Fig. 1).

We note that all observed events will lie within the area *OPQR* which is neither a tabulation by age and period (a $\square $ in a Lexis diagram), by period and cohort (a  in a Lexis diagram), or by age and cohort (a  in a Lexis diagram). If we are only interested in what happens until age *t*, then we must restrict ourselves to events lying in the area *OPQXV*.

By design, individuals within the same strata will have different length of follow-up. This can be overcome by simply dropping any events lying in the grey, hatched triangles in the Lexis diagram. While this is not strictly necessary in order to use the Kaplan–Meier or Aalen–Johansen estimator within each stratum, by doing so, we make sure not to extrapolate beyond the end of follow-up date *D*. We also see that, in the case of no loss-to-follow-up censoring, the Kaplan–Meier estimator reduces to a simple binomial estimator, see (6). Note however that the wider the strata the more events are dropped by this procedure so that this may not be the best option if strata are too wide.

If, after removal, we are only interested in what happens until age *t* that leaves us with the red, hatched area. The common end of follow-up age in the *i*th stratum after removal is denoted *t*_*i*_. We note that the available data for each stratum is thus tabulated by cohort and age () and that there is no administrative censoring within each stratum.

### A pooled analysis

The distribution of *T* may be characterized by the cumulative incidence function, CI(*t*)=*P*(*T*≤*t*), which is the probability of seeing an event before or at time *t*. Since CI(*t*)=1−*S*(*t*) with *S*(*t*)=*P*(*T*>*t*) being the survival function, the cumulative incidence at time *t* is usually estimated by $\widehat {\text {CI}}_{\mathrm {p}}(t)=1-\widehat {S}_{\mathrm {p}}(t)$, where

1 $$\begin{array}{*{20}l} \widehat{S}_{\mathrm{p}}(t)=\prod_{j:s_{j}\leqslant t}\frac{Y(s_{j})-d(s_{j})}{Y(s_{j})},\quad 0\leqslant t\leqslant t_{1},  \end{array} $$

is the (pooled) estimator obtained by applying the Kaplan–Meier estimator to the total sample. Here (*s*_*j*_) are the distinct event times, *Y*(*s*) the number at risk at time *s*, and *d*(*s*) the number of events at time *s* in the total sample, respectively. This pooled Kaplan–Meier estimator is unbiased for CI(*t*) for all *t* provided that censoring is independent. Often the cumulative incidence at the largest observed time, $\widehat {\text {CI}}_{\mathrm {p}}(t_{1})$, is reported as a summary measure of the overall event risk.

Generally speaking, the quantity $\widehat {\text {CI}}_{\mathrm {p}}(t)$ will be an estimate of the event probability at time *t* for a hypothetical individual that, at any given time point *s*≤*t*, has the same instantaneous risk of the event as those individuals still at risk in the study. However, under calendar time trends, the random variables *T* and *C* are not independent and hence it must be considered doubtful that the uncensored individuals should be representative for the target population. In fact, in the next section, we show that in a proportional hazards model, the pooled Kaplan–Meier estimator is indeed biased for CI(*t*) for certain *t*.

Let us further illustrate this point by considering the at-risk set depicted in the Lexis diagram of Fig. 1. In the first period [ 0,*t*_*k*_], members from all strata will be at risk, in the period (*t*_*k*_,*t*_*k*−1_] members from the first *k*−1 strata will be at risk, and so forth. In the last period (*t*_2_,*t*_1_] only members of the first stratum will be at risk. Thus, beyond time *t*_*k*_ the individuals still at risk comprise a selected group of individuals that, due to calendar time trends, are not representative for the target population.

### A proportional hazards analysis

An important message to convey is that when calendar time trends are present in a cohort study with staggered entry and administrative censoring, one cannot estimate the cumulative incidence beyond time *t*_*k*_ without imposing further assumptions. Such an assumption might be that hazard rates across strata are proportional, that is,

$$\begin{array}{*{20}l} \lambda_{i}(t)=\lambda_{1}(t)\exp(\beta_{i}),\quad 0\leqslant t\leqslant t_{1}, \end{array} $$

where *i*=2,…,*k* denotes the stratum. Assuming this model, standard statistical software can produce estimates $\widehat {S}_{1}(t)$ of the baseline survival function $S_{1}(t)=\exp \left (-{\int _{0}^{t}}\lambda _{1}(s)\,\text {ds}\right)$ for *t*≤*t*_1_ as well as estimates $\widehat {\beta }_{2},\ldots,\widehat {\beta }_{k}$ of the parameters. An (approximately) unbiased estimate of the cumulative incidence CI(*t*) in the target population may then be obtained by extrapolating each stratum’s estimated event probability at time *t* and take a weighted average: $\widehat {\text {CI}}_{\text {PH}}(t)=1-\widehat {S}_{\text {PH}}(t)$ with

2 $$\begin{array}{*{20}l} \widehat{S}_{\text{PH}}(t)=\sum_{i=1}^{k}\frac{n_{i}}{n}\widehat{S}_{1}(t)^{\exp(\hat{\beta}_{i})},\quad 0\leqslant t\leqslant t_{1},\quad \hat{\beta}_{1}=0. \end{array} $$

**Fig. 2:**
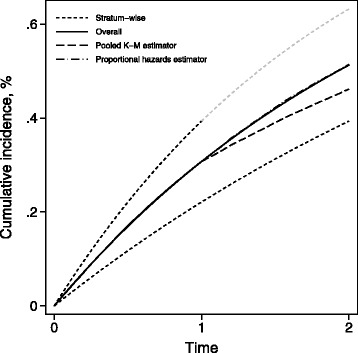
Cumulative incidence in a hypothethical cohort divided into two strata with a time trend given by a proportional hazards model. In the most prevalent stratum, an administrative censoring is imposed at time *t*=1 but no censoring is happening in the least prevalent stratum. Based on a sample of 50,000 individuals in each stratum, the pooled Kaplan–Meier estimator and the Kaplan–Meier estimator based on a proportional hazards model is computed

Figure 2 shows the cumulative incidence in a hypothetical cohort divided into two strata of equal size. The cumulative incidence is assumed to follow that of an exponential distribution with parameters 0.25 and 0.50 respectively. We assume that all individuals in the first cohort are followed until time *t*=2 whereas all individuals in the second cohort are subject to administrative censoring at time *t*=1 (indicated by the change in brightness of its cumulative incidence curve). The plot shows the theoretical cumulative incidence for both strata individually as well as combined (solid curve) and based on a sample of 50,000 individuals in each cohort, we show the pooled Kaplan–Meier estimator $\widehat {\text {CI}}_{\mathrm {p}}(t)$ and the estimator based on proportional hazards $\widehat {\text {CI}}_{\text {PH}}(t)$. The pooled Kaplan–Meier estimator is clearly seen to be biased for CI(*t*) for *t*>1 as a consequence of the censoring and of the time trend (between strata) whereas the $\widehat {\text {CI}}_{\text {PH}}(t)$ is on point.

The proportionality assumption may be checked e.g. by inspecting log-minus-log plots. However, the assumption can only be checked for *t*≤*t*_*i*_ in the *i*th stratum so that beyond time *t*_*i*_ we will have to rely on the assumption that the proportionality continues. For rare events, the assumption of proportional rates is equivalent to the assumption of no acceleration or deceleration in the event distribution. For autism it has been shown that the age at diagnosis has decreased over time [18] corresponding to an acceleration in the events distribution and as such, a proportional hazards model may not be the best option. Keep in mind that any significant deviation from proportionality will render the cumulative incidence estimate $\widehat {\text {CI}}_{\text {PH}}(t)$ invalid.

### A stratified analysis

When the proportional hazards assumption is unreasonable, we advocate for presenting the results from a stratified analysis. Let *S*_*i*_(*t*)=*P*(*T*>*t*∣*B*=*i*) denote the survival function at time *t* in the *i*th stratum. Since we have independent censoring within each stratum, we may unbiasedly estimate *S*_*i*_(*t*) by the Kaplan–Meier estimator in the *i*th stratum

3 $$\begin{array}{*{20}l} \widehat{S}_{i}(t)=\prod_{j:s_{ij}\leqslant t} \frac{Y_{i}(s_{ij})-d_{i}(s_{ij})}{Y_{i}(s_{ij})},\quad 0\leqslant t\leqslant t_{i},  \end{array} $$

where (*s*_*ij*_) are the distinct event times, *Y*_*i*_(*s*) the number at risk at time *s*, and *d*_*i*_(*s*) the number of events at time *s* in the *i*th stratum, respectively.

A stratified analysis would consist of arguing the validity of the independent censoring assumption within strata and presenting the estimated cumulative incidence curves ($t\mapsto \widehat {\text {CI}}_{i}(t)$) with $\widehat {\text {CI}}_{i}(t)=1-\widehat {S}_{i}(t)$ for *i*=1,…,*k*. The results may be simplified by presenting just the end of follow-up cumulative incidence estimates across strata $\widehat {\text {CI}}_{1}(t_{1}),\ldots,\widehat {\text {CI}}_{k}(t_{k})$ (or even simpler just their range) alongside the (range of) end of follow-up times across strata *t*_1_,…,*t*_*k*_. Later we will see how these end of follow-up estimates may be combined into an estimate of the cumulative incidence within the study period.

### Competing risks

The Kaplan–Meier estimator should only be used when there are no or minimal competing risks but this is not always the case. For instance, if one is interested in the disease risk in adulthood it may be errorenous to ignore death as a competing risk. In this case, if *h* corresponds to the event of interest then the cause-*h*-specific cumulative incidence at time *t* in the *i*th stratum should instead be estimated by the Aalen–Johansen estimator [1]

4 $$\begin{array}{*{20}l} \widehat{\text{CI}}_{ih}(t)=\sum_{j:s_{ij}\leqslant t}\frac{\widehat{S}_{i}(s_{ij}-)}{Y_{i}(s_{ij})}\mathbf{1}_{D_{ij}=h},\quad 0\leqslant t\leqslant t_{i},  \end{array} $$

where *D*_*ij*_ is the cause at time *s*_*ij*_. The estimator is approximately unbiased for the cause-*h*-specific cumulative incidence in the *i*th stratum when the censoring is independent in the *i*th stratum [3].

## Results

### Example 1: Examining calendar time trends in psychiatric disorders

A stratified analysis was used in [4] to examine calendar time trends in four psychiatric disorders in Denmark: autism spectrum disorder (ASD), hyperkinetic disorder (HKD), obsessive-compulsive disorder (OCD), and Tourette syndrome (TS). The authors compared estimated cumulative incidence curves across strata given by 2-year birth cohorts taking emigration to other countries into account (loss-to-follow-up censoring). The analysis was based on all individuals born 1990–1999 in Denmark with end of follow-up on December 31, 2004. Graphical inspection of the cumulative incidence curves revealed an increasing time trend in three (ASD, HKD, and TS) of the four disorders. The study showed that the known increase in reported autism diagnoses at that time was not unique among childhood neuropsychiatric disorders but was part of a more widespread epidemiological phenomenon.

We will now re-examine time trends in the same four psychiatric disorders as studied in [4] using more birth years and a longer follow-up period than they did. At the same time we will demonstrate how different the curve obtained by the pooled estimator can be compared to the cumulative incidence curves obtained by stratification. To do so, we consider our sample consisting of all births in Denmark in the period 1980–2007 with end of follow-up on December 31, 2013. Compared to the data used in [5], our sample consists of 18 additional birth years as well as 9 years of additional follow-up time. Individuals are monitored from birth until diagnosis, emigration, death, or the end of follow-up, whichever occurs first. In particular, the largest observed age is 34 years. Emigration is treated as a censoring while death is considered a competing event. We let strata be given by 1-year birth cohorts (*k*=28 strata) and let the common end of follow-up ages be *t*_1_=33,*t*_2_=32,…,*t*_28_=6. By doing so, we make sure that all individuals within the same stratum potentially have the same length of follow-up (this corresponds to disregarding events in the grey, hatched triangles of Fig. 1).

**Fig. 3:**
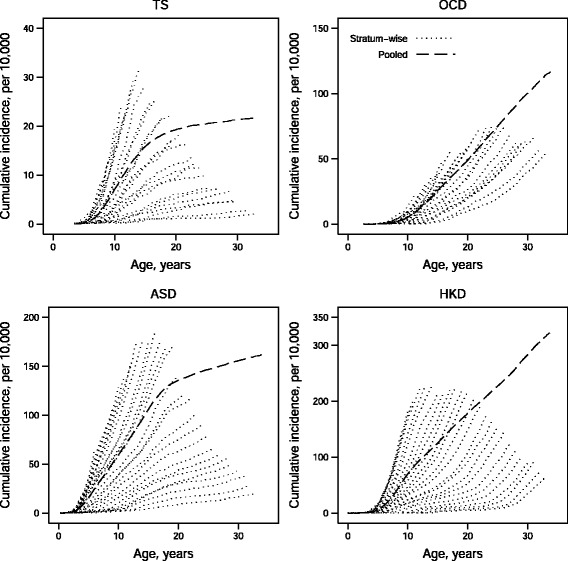
Age-specific cumulative incidence estimates for four psychiatric disorders using stratification (1-year birth cohorts) and using the pooled estimator. For both methods, the Aalen–Johansen estimator was used to account for competing risks. *TS*: Tourette syndrome; *OCD*: obsessive-compulsive disorder; *ASD*: autism spectrum disorder; *HKD*: hyperkinetic disorder

In Fig. 3, we have estimated the age-specific cumulative incidence for each strata and each disorder using the Aalen–Johansen estimator to account for death as a competing risk. The figure also shows the pooled Aalen–Johansen estimator obtained by applying the Aalen–Johansen estimator to the total sample ignoring the issue of dependent censoring. We see a clear time trend in all four disorders consistent with [4]. We also note that the pooled estimator for HKD and OCD near the end of follow-up exceeds all stratum-wise cumulative incidence curves. This reflects the fact that near the end of follow-up, the behaviour of the pooled estimator is dictated by the behaviour in the oldest cohorts. For this pooled estimate to be a valid estimate of the average cumulative incidence across all 28 cohorts, the cumulative incidence in the younger cohorts must continue to rapidly increase in the remaining period. This might or might not be a reasonable assumption, however, if we believe that the early rise in cumulative incidence in the younger cohorts are mainly due to a decrease in the mean age at diagnosis, then one would expect the incidence curves in the younger cohorts to flatten out at some point.

If we are not interested in presenting all 28 individual cumulative incidence curves, we might simply say that the cumulative incidence for e.g. HKD ranged between 37–229 per 10,000 at ages 6–33 for births 1980–2007. Taking the weighted average of the end of follow-up estimates yields an estimate of the cumulative incidence within the study period of 182 per 10,000 for HKD. This weighted average as an estimator is discussed in the Results section but for now we just note that it is much lower than the pooled estimate of 326 per 10,000. Consequently, the pooled estimator is seen not to be a valid estimate of the cumulative incidence within the study period either.

There is however a choice to be made in the number of strata to use. The key here is to remember that the wider strata the more data is disregarded in the analysis – recall that data in the grey, hatched triangles of Fig. 1 is not used. In this example, using 1-year birth cohorts amounts to excluding roughly 5% of all events. Thus, it is our recommendation to use relatively narrow strata chosen such that a further sub-division does not alter the results significantly. In our example, going from 1-year birth cohorts to 6-month birth cohorts changed the range from 37–229 per 10,000 to 38–251 per 10,000. Since the difference is small and has no effect on our conclusions, we feel confident presenting the results obtained with 1-year birth cohorts.

### Example 2: Cross-country comparisons of the risk of psychiatric disorders

In a later study, calendar time trends of the same four psychiatric disorders were re-examined with additional birth years and longer follow-up time [5]. In addition, the authors wanted to compare risks across four countries (Denmark, Sweden, Finland, and Western Australia). In this study, the authors now considered births in the period 1990–2007 with end of follow-up on December 31, 2011, and the outcome being either ASD, HKD, OCD, or TS for all countries. Their main analysis was a stratified analysis with strata given by 3-year birth cohorts resulting in 6 strata for each disorder and each country. That is, the analysis yielded 6 estimated cumulative incidence curves for each country and each disorder.

To simplify things we will here focus on comparing risks between Denmark and Finland. Comparing the risk of one psychiatric disorder in Denmark and Finland would consist of comparing the estimated cumulative incidence curves for each stratum. A simpler comparison would be to compare just the end of follow-up cumulative incidence estimates within each stratum. In the present example, this corresponds to 6 pairwise comparisons. Table 1 shows the estimated end of follow-up cumulative incidences per 10,000 for each of the four disorders and each stratum in Denmark and Finland (read of Supplementary Table 1 of [5]). For ASD, OCD and TS there is a clear tendency that these disorders are more commonly diagnosed in Denmark compared to Finland since cumulative incidence estimates are larger in Denmark compared to Finland for all strata. For HKD a tendency is less clear since it is more commonly diagnosed in Finland in two of the six strata and more commonly diagnosed in Denmark in the remaining four strata. In the light of this, it may be of interest to make an even simpler comparison of the disease risk in the two countries based on what is observed within the study period.

**Table 1:** End of follow-up cumulative incidence estimates per 10,000 in strata given by 3-year birth cohorts for four psychiatric disorders in Denmark and Finland based on 1990–2007 births with end of follow-up on December 31, 2011

		Birth cohort strata					
Country	Disorder	90–92	93–95	96–98	99–01	02-04	05–07
Denmark	TS	32.1	35.1	46.3	31.2	16.5	1.6
	OCD	57.3	32.1	40.3	10.8	4.5	0.4
	ASD	109.7	117.0	160.3	110.3	88.5	35.5
	HKD	164.6	150.5	222.2	168.0	109.9	19.0
Finland	TS	9.8	10.1	15.1	8.9	5.3	0.3
	OCD	38.2	17.4	26.4	4.3	1.6	0.1
	ASD	77.1	83.3	101.5	59.8	51.7	26.0
	HKD	133.1	159.6	225.6	142.3	100.5	11.6

*TS* Tourette syndrome, *OCD* obsessive-compulsive disorder, *ASD* autism spectrum disorder, *HKD* hyperkinetic disorder. Data is from the Supplementary Table 1 of [5]

### The event risk within the study period

When strata are many, we might be interested in comparing a single summary measure of the disease risk to assess differences across countries. We have seen that it is not possible to estimate the cumulative incidence beyond age *t*_*k*_ under time trends without imposing further assumptions. It is however possible to estimate the risk of seeing an event before a specific time and before the end of follow-up. This measure may be useful to compare event risks across different countries if based on studies with similar follow-up. We will call this summary measure *the event risk within the study period*. Before defining this summary measure we will introduce the stratified Kaplan–Meier estimator discussed in [2].

By the law of total probability we may write *S*(*t*) as a weighted average of stratum-specific survival functions

$$\begin{array}{*{20}l} S(t)=\sum_{i=1}^{k}\pi_{i} S_{i}(t),\quad t\geqslant 0. \end{array} $$

Thus, an approximately unbiased estimate of *S*(*t*) can only be given in the time interval up to the smallest end of follow-up time *t*_*k*_:

$$\begin{array}{*{20}l} \widehat{S}_{\mathrm{s}}(t)=\sum_{i=1}^{k} \frac{n_{i}}{n}\widehat{S}_{i}(t),\quad 0\leqslant t\leqslant t_{k}. \end{array} $$

Since it is only well-defined up to time *t*_*k*_, this estimator will often be of limited practical use. In our example with 1980–2007 births, administrative censoring at the end of 2013, and strata given by each birth year, this estimator only allows us to unbiasedly estimate the age-specific cumulative incidence until the age of 6.

Consider instead the weighted average of the stratum-wise Kaplan–Meier estimates truncated at the end of follow-up

5 $$\begin{array}{*{20}l} \widehat{S}_{\mathrm{w}}(t)=\sum_{i=1}^{k} \frac{n_{i}}{n}\widehat{S}_{i}(t\wedge t_{i}),\quad 0\leqslant t\leqslant t_{1},  \end{array} $$

and let $\widehat {\text {CI}}_{\mathrm {w}}(t)=1-\widehat {S}_{\mathrm {w}}(t)$. We will call this the Kaplan–Meier estimator of the event risk at time *t* within the study period. We note that until time *t*_*k*_, this estimator is identical to the stratified estimator, i.e., $\widehat {S}_{\mathrm {w}}(t)=\widehat {S}_{\mathrm {s}}(t)$ for *t*≤*t*_*k*_. This reflects the fact that the administrative censoring does not occur before time *t*_*k*_.

Let $U=\sum _{i=1}^{k} t_{i}\mathbf {1}_{B=i}$ denote the end of follow-up variable based on the *k* strata given by *B* and let CI^′^(*t*)=*P*(*T*≤*t*∧*U*) be the probability of seeing an event before time *t* *and* before the end of follow-up. The within-study estimator $\widehat {\text {CI}}_{\mathrm {w}}(t)$ is seen to be (approximately) unbiased for CI^′^(*t*) for any *t*≤*t*_1_ (see Additional file 1). We emphasize that CI^′^(*t*) does not have an interpretation applying directly to the uncensored target population since its interpretation does involve the end of follow-up variable *U*. In terms of the Lexis diagram of Fig. 1, we see that $\widehat {\text {CI}}_{\mathrm {w}}(t)$ will estimate the probability that an individual entering the study between *O* and *P* has an event in the red, hatched area.

Let *e*_*i*_(*t*) denote the number of events before time *t* available in the *i*th stratum after removal of data in the grey, hatched triangles of Fig. 1. In particular, $\sum _{i=1}^{k} e_{i}(t)$ will be the number of events in the red, hatched area of Fig. 1. In the case of no loss-to-follow-up censoring we then have

6 $$\begin{array}{*{20}l} \widehat{\text{CI}}_{\mathrm{w}}(t)=\frac1n\sum_{i=1}^{k} e_{i}(t),\quad 0\leqslant t\leqslant t_{1},  \end{array} $$

meaning that, in this case, the within-study estimator is a simple binomial estimator.

Note also that we can write

7 $$\begin{array}{*{20}l} \widehat{\text{CI}}_{\mathrm{w}}(t_{1})=\sum_{i=1}^{k} \frac{n_{i}}{n}\widehat{\text{CI}}_{i}(t_{i})  \end{array} $$

meaning that $\widehat {\text {CI}}_{\mathrm {w}}(t_{1})$ is simply the weighted average of the end of follow-up cumulative incidence estimates introduced above. This will be an (approximately) unbiased estimate of CI^′^(*t*_1_)=*P*(*T*≤*U*) which we will refer to as the event risk within the study period. This is the probability that an individual entering between *O* and *P* has an event in the area given by *OPQR* minus the six grey, hatched triangles in our Lexis diagram (Fig. 1). When the removal of the grey, hatched triangles is of little significance (i.e., when strata are so narrow that the triangles hold few events), we may say that $\widehat {\text {CI}}_{\mathrm {w}}(t)$ estimates the event risk before end of follow-up on date *D* corresponding to the probability of an event in the area *OPQR*. Thus, in our example, $\widehat {\text {CI}}_{\mathrm {w}}(t_{1})$ will estimate the risk of getting a diagnosis before December 31, 2013, for individuals born in the period 1980–2007.

The within-study estimator $\widehat {\text {CI}}_{\mathrm {w}}(t)$ has the following advantages: 1) it has a clear and useful interpretation and 2) it can be unbiasedly estimated in a study with staggered entry, administrative censoring and calendar time trends. Moreover, it allows for an easier cross-country comparison of event risks than doing *k* pairwise comparisons. For this comparison to make sense it is however important that the country-specific analyses are all based on roughly the same years of entry, have roughly the same end of follow-up date and that strata are of similar relative size.

### The variance of the Kaplan–Meier estimator of the event risk within the study period

For the estimator in (7) to be of any use we need the ability to compute confidence intervals. An estimator of the variance of $\widehat {S}_{\mathrm {w}}(t_{1})=1-\widehat {\text {CI}}_{\mathrm {w}}(t_{1})$ is given by (see Additional file 1)

8 $$\begin{array}{*{20}l} \widehat{\text{Var}}(\widehat{S}_{\mathrm{w}}(t_{1}))&=\frac{1}{n}\left[\sum_{i=1}^{k}\frac{n_{i}}{n}\widehat{S}_{i}(t_{i})^{2}-\left(\sum_{i=1}^{k} \frac{n_{i}}{n}\widehat{S}_{i}(t_{i})\right)^{2}\right]\\ &\quad +\sum_{i=1}^{k}\frac{{n_{i}^{2}}}{n^{2}}\widehat{\text{Var}}(\widehat{S}_{i}(t_{i})),  \end{array} $$

where

$$\begin{array}{*{20}l} \widehat{\text{Var}}(\widehat{S}_{i}(t_{i}))=\widehat{S}_{i}(t_{i})\sum_{s_{ij}\leqslant t_{i}}\frac{d_{ij}}{Y_{i}(s_{ij})[\!Y_{i}(s_{ij})-d_{ij}]} \end{array} $$

is Greenwood’s formula applied to the *i*th stratum [11].

An estimator for the variance of the competing risks counterpart of (7) can be obtained by replacing Greenwood’s estimate in (8) by an estimator for the variance of the Aalen–Johansen estimator in the *i*th stratum (see e.g. [14]).

### Example 2 (cont.): Comparing the risk of psychiatric disorders across countries

Let us briefly return to the example of comparing risks of psychiatric disorders in Denmark and Finland. Assuming that the number of births per year are constant in the period 1990–2007 in both Denmark and Finland (this is roughly the case) we have that *n*_*i*_/*n*=1/6 for *i*=1,…,6 in both countries. In this case the Kaplan–Meier estimator of the risk within the study period for each disorder and each country is simply the average of the rows in Table 1.

For TS we obtain an estimate of 27.1 per 10,000 in Denmark compared to 8.3 per 10,000 in Finland. These estimates has the interpretation as the average risk of getting a TS diagnosis before December 31, 2011, for children born 1990–2007 in Denmark and Finland respectively. Earlier we saw that for HKD it was not clear if the disorder was more commonly diagnosed for children born in Denmark or Finland in the period 1990–2007. The estimator of the risk within the study period was for HKD 139.0 per 10,000 in Denmark and 128.8 per 10,000 in Finland suggesting that it is slightly more common in Denmark within the study period. These estimates should of course be judged by a confidence interval but these could not be computed as we do not have access to the original data.

## Discussion

Studies with staggered entry and administrative censoring are very common within health sciences. A classic example of this design is when the event of interest is the occurence of some disease or disorder in individuals followed from birth until a fixed end of follow-up date. Another classic example is when patients diagnosed with and possibly treated for a certain disease are monitored for relapse or death before a specific date.

In the examples above, we did a stratified analysis with strata given by time of entry into the study and the administrative censoring within strata were dealt with by excluding all events in the grey, hatched triangles of Fig. 1. For the four disorders in question that corresponds to excluding about 5% of the total number of events. Excluding events is usually not advisable but in this particular setting it has its merits. First, we make sure not to extrapolate beyond the end of follow-up date *D*. Second, we see that, in the absence of loss-to-follow-up censoring, the within-study estimator reduces to a simple binomial estimator. Of course, one should be careful not to choose strata too wide since the wider the strata the more events are excluded. However, this is something that can be controlled by the analyst and if the analyst is unhappy about the number of events dropped, he or she can simply narrow down the strata until satisfied.

Issues connected to calendar time trends are well-known in demography where lifetime expectancy and fertility rates are commonly estimated [6, 12]. Both lifetime expectancy and fertility are measures that are and have been under the influence of time trends, for instance, mortality rates have decreased over time due to improving health care systems and better diagnostic tools over the years. Estimates of lifetime expectancy and fertility rates are based on data tabulated by period and age corresponding to e.g. the dashed, vertical rectangle in Fig. 1. It is however our impression that outside of demography issues connected to calendar time trends are lesser known.

A reason that a pooled analysis using the Kaplan–Meier estimator is so widely used is most likely that the analysis is easy to carry out and the results are easily presented; for a single outcome, the results from a pooled analysis is either a curve or maybe just a few points on the curve. Another reason may be a belief that if the disease risk varies across certain strata, then a pooled analysis will yield an estimate of the average age-specific cumulative incidence across strata. This property holds for the simple binomial estimator with complete follow-up but it does not hold in a time-to-event setting whenever the censoring mechanism is not independent. The general advice is thus to only do a pooled analysis if one is certain that the censoring mechanism is independent.

## Conclusions

Disease risk estimates may affect decision-making within health care in general but also affect which diseases or disorders that have research funding allocated to them. As such, it is essential that we are able to correctly estimate disease risks. In this paper, we argue that, for certain study designs, the usual pooled estimators should be avoided whenever the disease risk is influenced by calendar time trends. We present some alternative methods that provide non-inflated estimates of the disease risk.

More specifically, we argue that censoring is not independent in studies with staggered entry and administrative censoring whenever calendar time trends are present in the disease risk. Since the pooled Kaplan–Meier and Aalen–Johansen estimators rely heavily on this assumption, we advocate against using these whenever a disease is influenced by time trends. Instead we suggest using a proportional hazards model to extrapolate the age-specific cumulative incidence if proportionality is a reasonable assumption. If not, we suggest presenting the results from a stratified analysis where strata are chosen such that administrative censoring is not an issue within strata.

It may be difficult to present the results from a stratified analysis concisely when strata are many. If one is interested in a concise way of describing the general disease risk in a population with calendar time trends, we suggest using the within-study estimator. This will estimate the general risk of the disease within the study period in question. We emphasize that the within-study estimator depends on the recruitment period (e.g. birth years), the end of follow-up date and the choice of strata and thus, for it to serve as a way comparing disease risks across countries, the country-specific studies should be based on roughly the same recruitment period, have roughly the same end of follow-up and use roughly the same stratification.

## Additional file

Additional file 1 The variance of the Kaplan–Meier estimator of the event risk within the study period. (PDF 211 kb)

## Abbreviations

ASD: Autism spectrum disorder
CI: Cumulative incidence
HKD: Hyperkinetic disorder
OCD: Obsessive-compulsive disorder
TS: Tourette syndrome
