# Supplementary material for: Estimating a population cumulative incidence under calendar time trends
Source: BMC Med Res Methodol. 2017 Jan 11;17:7. doi: 10.1186/s12874-016-0280-6 (PMC5225659; doi:10.1186/s12874-016-0280-6)
Supplement: Additional file 1 — The variance of the Kaplan–Meier estimator of the event risk within the study period. (PDF 211 kb) [file 12874_2016_280_MOESM1_ESM.pdf]

Estimating a population cumulative incidence under calendar time trends

Stefan N. Hansen*[1], Morten Overgaard[1], Per K. Andersen[2], and Erik T. Parner[1]

[1] *Section for Biostatistics, Aarhus University*
[2] *Section of Biostatistics, University of Copenhagen*

BMC Medical Research Methodology

Additional file 1

The variance of the Kaplan–Meier estimator of the event risk within the study period

We derive the weighted average of the stratum-wise Kaplan–Meier estimators as a non-parametric maximum likelihood estimator and its asymptotic variance when the π_i's are considered unknown.

Suppose we have a sample consisting of n_i observations from the ith stratum with $n = \sum_i n_i$ being the total sample size. Due to right-censoring we observe $t_{ij} = \tilde{t}_{ij} \wedge c_{ij}$, where $\tilde{t}_{ij}$ is the actual time-to-event and c_{ij} is the censoring time, and $\delta_{ij} = \mathbf{1}_{\tilde{t}_{ij} \leqslant c_{ij}}$ for $i = 1, \ldots, k$ and $j = 1, \ldots, n_i$ with observations being independent. Under independent right-censoring the likelihood is

$$\begin{aligned} L(\pi_1, \ldots, \pi_k, S_1, \ldots, S_k) &= \prod_{i=1}^{k} \prod_{j=1}^{n_i} \pi_i f_i(t_{ij})^{\delta_{ij}} S_i(t_{ij})^{1-\delta_{ij}} \\ &= \prod_{i=1}^{k} \pi_i^{n_i} \prod_{j=1}^{n_i} f_i(t_{ij})^{\delta_{ij}} S_i(t_{ij})^{1-\delta_{ij}} \end{aligned}$$

with $(S_1, \ldots, S_k)$ and $(f_1, \ldots, f_k)$ being the conditional survival functions and conditional densities in the k strata respectively and $(\pi_1, \ldots, \pi_k)$ being the probabilities of belonging to the strata. The corresponding non-parametric likelihood function [2] is thus

$$\widetilde{L}(\pi_1, \ldots, \pi_k, S_1, \ldots, S_k) = \prod_{i=1}^{k} \pi_i^{n_i} \prod_{j=1}^{n_i} \Delta S_i(t_{ij})^{\delta_{ij}} S_i(t_{ij})^{1-\delta_{ij}}$$

with $\Delta S_i(t_{ij}) = S_i(t_{ij}-) - S_i(t_{ij})$ being the jump at t_{ij}. Maximizing this non-parametric likelihood function yields the estimates $\widehat{\pi}_i = n_i/n$ which is the observed proportion and $\widehat{S}_i = (\widehat{S}_i(t))_{t \geq 0}$, the Kaplan–Meier estimator in the ith stratum, for $i = 1, \ldots, k$.

Let $S_{\mathrm{w}}(t_1) = P(T \leqslant U)$ with $U = \sum_{i=1}^{k} t_i \mathbf{1}_{B=i}$ and let

$$\widehat{S}_{\mathrm{w}}(t_1) = \sum_{i=1}^{k} \frac{n_i}{n} \widehat{S}_i(t_i)$$

denote the Kaplan–Meier estimator of the within-study event risk. If $\widehat{\boldsymbol{\pi}} = (\widehat{\pi}_1, \ldots, \widehat{\pi}_k)^\intercal$ is the vector of observed proportions and $\widehat{\mathbf{S}} = (\widehat{S}_1(t_1), \ldots, \widehat{S}_k(t_k))^\intercal$ is the vector of Kaplan–Meier estimates evaluated at the end of follow-up times $t_1, \ldots, t_k$, then by properties of the non-parametric maximum likelihood estimates we have

$$\sqrt{n}\Big(\begin{pmatrix} \widehat{\boldsymbol{\pi}} \\ \widehat{\mathbf{S}} \end{pmatrix} - \begin{pmatrix} \boldsymbol{\pi} \\ \mathbf{S} \end{pmatrix} \Big) \xrightarrow{\mathcal{D}} \mathcal{N}_{2k}\Big(\mathbf{0}, \begin{pmatrix} \boldsymbol{\Sigma}_1 & \mathbf{0} \\ \mathbf{0} & \boldsymbol{\Sigma}_2 \end{pmatrix}\Big),$$

*Corresponding author; Address: Bartholins Allé, DK-8000 Aarhus C – E-mail: stefanh@ph.au.dk

where $\boldsymbol{\pi} = (\pi_1, \dots, \pi_k)^\intercal$, $\mathbf{S} = (S_1(t_1), \dots, S_k(t_k))^\intercal$. Here

$$\boldsymbol{\Sigma}_1 = \mathrm{diag}(\boldsymbol{\pi}) - \boldsymbol{\pi}\boldsymbol{\pi}^\intercal, \qquad \boldsymbol{\Sigma}_2 = \mathrm{diag}(\sigma_1^2/\pi_1, \dots, \sigma_k^2/\pi_k),$$

for some $\sigma_1^2, \dots, \sigma_k^2 > 0$.

The delta method [1] on $g(x_1, \dots, x_{2k}) = \sum_{i=1}^k x_i x_{k+i}$ now yields

$$\sqrt{n}\big(\widehat{S}_{\mathrm{w}}(t_1) - S_{\mathrm{w}}(t_1)\big) \xrightarrow{\mathcal{D}} \mathcal{N}_1(0, \sigma_{\mathrm{w}}^2)$$

with

$$\sigma_{\mathrm{w}}^2 = \begin{pmatrix} \mathbf{S}^\intercal & \boldsymbol{\pi}^\intercal \end{pmatrix} \begin{pmatrix} \boldsymbol{\Sigma}_1 & \mathbf{0} \\ \mathbf{0} & \boldsymbol{\Sigma}_2 \end{pmatrix} \begin{pmatrix} \mathbf{S} \\ \boldsymbol{\pi} \end{pmatrix} = \mathbf{S}^\intercal \boldsymbol{\Sigma}_1 \mathbf{S} + \boldsymbol{\pi}^\intercal \boldsymbol{\Sigma}_2 \boldsymbol{\pi}.$$

The variance of this Kaplan–Meier estimator is thus approximately given by

$$\mathrm{Var}(\widehat{S}_{\mathrm{w}}(t_1)) \approx \frac{\sigma_{\mathrm{w}}^2}{n} = \frac{1}{n}\left[\sum_{i=1}^k \pi_i S_i(t_i)^2 - \Big(\sum_{i=1}^k \pi_i S_i(t_i)\Big)^2 + \sum_{i=1}^k \pi_i \sigma_i^2\right].$$

If $\widehat{\mathrm{Var}}(\widehat{S}_i(t_i))$ is Greenwood's estimate of the variance σ_i^2/n_i of the Kaplan–Meier estimator in the ith group, then σ_i^2 can be estimated by $n_i \widehat{\mathrm{Var}}(\widehat{S}_i(t_i))$. Thus, we may estimate the variance by

$$\widehat{\mathrm{Var}}(\widehat{S}_{\mathrm{w}}(t_1)) = \frac{1}{n}\left[\sum_{i=1}^k \frac{n_i}{n}\widehat{S}_i(t_i)^2 - \Big(\sum_{i=1}^k \frac{n_i}{n}\widehat{S}_i(t_i)\Big)^2\right] + \sum_{i=1}^k \frac{n_i^2}{n^2}\widehat{\mathrm{Var}}(\widehat{S}_i(t_i)).$$

References

[1] Lehmann, E.L.: Elements of Large-Sample Theory. Springer-Verlag, New York (1999)

[2] van der Vaart, A.W.: Asymptotic Statistics. Cambridge University Press, Cambridge (2000)
